# Optimizing COVID-19 surveillance using historical electronic health records of influenza infections

**DOI:** 10.1093/pnasnexus/pgac038

**Published:** 2022-04-14

**Authors:** Zhanwei Du, Yuan Bai, Lin Wang, Jose L Herrera-Diestra, Zhilu Yuan, Renzhong Guo, Benjamin J Cowling, Lauren A Meyers, Petter Holme

**Affiliations:** Shenzhen Institute of Research and Innovation, The University of Hong Kong, Shenzhen 518057, China; World Health Organization Collaborating Centre for Infectious Disease Epidemiology and Control, School of Public Health, University of Hong Kong, Hong Kong SAR 999077, China; Laboratory of Data Discovery for Health, Hong Kong Science Park, Hong Kong SAR 999077, China; The University of Texas at Austin, Austin, TX 78712, USA; World Health Organization Collaborating Centre for Infectious Disease Epidemiology and Control, School of Public Health, University of Hong Kong, Hong Kong SAR 999077, China; Laboratory of Data Discovery for Health, Hong Kong Science Park, Hong Kong SAR 999077, China; University of Cambridge, Cambridge CB2 3EH, UK; The University of Texas at Austin, Austin, TX 78712, USA; Department of Biology, The Pennsylvania State University, University Park, PA 19104, USA; Research Institute for Smart Cities, School of Architecture and Urban Planning, Shenzhen University, Shenzhen 518060, China; Research Institute for Smart Cities, School of Architecture and Urban Planning, Shenzhen University, Shenzhen 518060, China; World Health Organization Collaborating Centre for Infectious Disease Epidemiology and Control, School of Public Health, University of Hong Kong, Hong Kong SAR 999077, China; Laboratory of Data Discovery for Health, Hong Kong Science Park, Hong Kong SAR 999077, China; The University of Texas at Austin, Austin, TX 78712, USA; Department of Computer Science, Aalto University, Espoo 00076, Finland; Center for Computational Social Science, Kobe University, Kobe 657-8501, Japan

**Keywords:** COVID-19, influenza, electronic health records, epidemiology, surveillance

## Abstract

Targeting surveillance resources toward individuals at high risk of early infection can accelerate the detection of emerging outbreaks. However, it is unclear which individuals are at high risk without detailed data on interpersonal and physical contacts. We propose a data-driven COVID-19 surveillance strategy using Electronic Health Record (EHR) data that identifies the most vulnerable individuals who acquired the earliest infections during historical influenza seasons. Our simulations for all three networks demonstrate that the EHR-based strategy performs as well as the most-connected strategy. Compared to the random acquaintance surveillance, our EHR-based strategy detects the early warning signal and peak timing much earlier. On average, the EHR-based strategy has 9.8 days of early warning and 13.5 days of peak timings, respectively, before the whole population. For the urban network, the expected values of our method are better than the random acquaintance strategy (24% for early warning and 14% in-advance for peak time). For a scale-free network, the average performance of the EHR-based method is 75% of the early warning and 109% in-advance when compared with the random acquaintance strategy. If the contact structure is persistent enough, it will be reflected by their history of infection. Our proposed approach suggests that seasonal influenza infection records could be used to monitor new outbreaks of emerging epidemics, including COVID-19. This is a method that exploits the effect of contact structure without considering it explicitly.

Significance Statement Studies of network epidemiology suggest that central nodes in a network are at a higher risk of being infected early in an outbreak and can, thus be identified as being disproportionately represented among previous infections. However, detailed data on interpersonal, physical contacts are difficult to obtain from most cities. Therefore, it is unclear which individuals would have a higher risk of early infection in the real world. Here, we develop a new COVID-19 surveillance approach that uses historical EHRs of influenza patients. Our method uses digital traces of influenza treatments to exploit the underlying structure of interpersonal contacts. Our proposed approach suggests that seasonal influenza treatment records can be used better to monitor new outbreaks of emerging epidemics, including COVID-19.

## Introduction

A novel coronavirus (SARS-CoV-2) is thought to have emerged in the last quarter of 2019 in Wuhan, China (1), and was declared a pandemic by the World Health Organization (WHO) on 2020 March 11 (2). By 2021 September 29, 219 million cases of COVID-19 and 4.6 million deaths (3) were reported worldwide. Infectious disease surveillance systems provide critical information on the occurrence of infections and allow early detection of COVID-19 outbreaks before they spread out of control. Surveillance of COVID-19 has relied mainly on reported cases, contact tracing, and projections (4, 5), coupled with syndromic surveillance systems to track anomalous increases in COVID-like-illness (CLI) symptoms (5, 6).

In the past decade, public health agencies have benefited from an influx of medical, epidemiological, and computational scientists, who have developed model-based capabilities for empirical analysis and mathematical modeling that capture the unfolding of epidemic outbreaks, their mechanisms, and their response strategies. However, the increasing frequency of unexpected emerging and reemerging infectious diseases demonstrates the need for improved capacity to accelerate outbreak detection in designing disease surveillance systems (7).

With more effective surveillance on more vulnerable individuals as potential infection reservoirs, we can more effectively uncover early signals of an emerging epidemic outbreak, allowing expedite and optimal deployment of resources for its control. Decades of epidemiology research have demonstrated the influence of the contact network structure on epidemic outbreaks by determining whether and when susceptible individuals are infected (7–12). The surveillance strategies that map out individual contact behaviors fall into those based on static contact networks (13). Retrospective research has designed pioneer strategies based on topological structures—for example, ref. (14) presented a simple social-network-based strategy (random acquaintance) in a college population by monitoring friends of randomly selected students as the random acquaintance surveillance group (SG). The random acquaintance SG is expected to exhibit a signal 2 weeks earlier than the random surveillance strategy (selection of the surveillance subset randomly from the population). Moreover, ref.^7^ further investigate different centrality-based surveillance strategies, showing how the complete knowledge of the network of social interactions can be used to propose strategies that outperform random and random-acquaintance strategies.

Medical, epidemiological, and computational scientists have recognized the promise of network-based outbreak detection to improve epidemic preparedness and response. However, few of these strategies are applied to practical public health systems due to the challenging implementation needed, additional huge workforce, and economic cost to explore the generally unknown contact network (15). The deluge of available digital data on Electronic Health Records (EHR) in public health systems offers unprecedented opportunities to explore novel sentinel surveillance strategies, which have been used for contact tracing in South Korea in the context of the COVID-19 epidemic (16, 17).

The aim of outbreak detection using sentinel surveillance is to detect a signal for the emerging outbreak as early as possible. This is similar to the well-studied problem of optimal vaccination on networks (18). Ref. (15) propose an innovative vaccination method targeting previously infected individuals as reported by individual infection history in EHRs. Previously infected individuals have a disproportionate probability of being highly connected within networks and transmitting to others. This targeted strategy is validated in contact network epidemiology simulations and confirmed by empirical clinical data from Israel (15).

The current study introduces a practical data-driven surveillance strategy to accelerate outbreak detection using the simple logic of targeting the earliest infected individuals by retrospective analysis of historical outbreaks. We assume that the latest influenza-like outbreaks would share closely similar networks of contacts as COVID-19 spreads in its early stages throughout the same region. Assuming that the past predicts the future in contact networks and that the past was affected by network structures, our method exploits the network structure without explicitly mapping out the contacts. In that sense, the method is network-free (even though the underlying processes are not).

Informed by historical influenza-like observations of individuals, we use mathematical epidemic models to systematically compare our proposed method with two well-studied surveillance strategies (e.g. random acquaintance and most connected) in the context of sentinel placement in networks where a COVID-19-like disease is spreading. We quantify the timing and accuracy of the information gained by these strategically chosen sensors, as well as the robustness in the selection of nodes with respect to the number of previous information (seasons) used and epidemiological outbreaks over different effective reproduction numbers, *R_e_*.

## Results

### Surveillance strategy using EHRs of historical influenza infections

We propose a new surveillance strategy that uses individuals estimated with high risks of having an early infection in a new outbreak. We assume that each individual who acquired influenza infection in a previous influenza season would have a digital record in the EHR system, providing key epidemiologic information, including the potential infection time. Considering the effect of short-term cross-strain immunity after an influenza infection (23), we assume that each individual can be infected at most once in a single influenza season. We assume that the EHR data are available for multiple seasons.

**Fig. 1.**
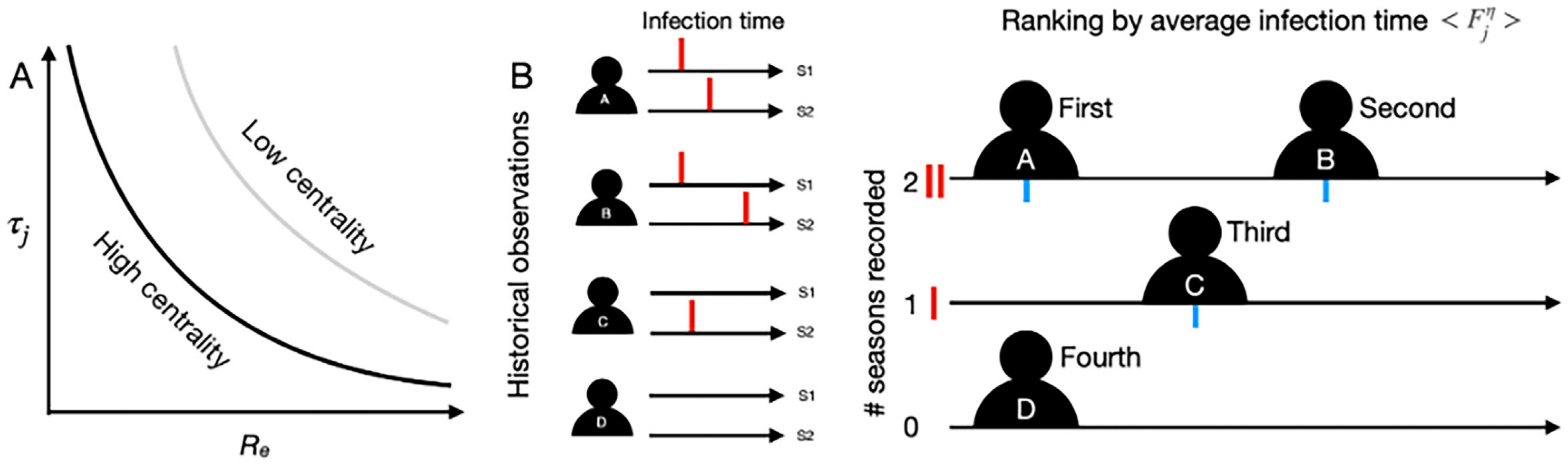
A schematic of the proposed surveillance strategy. (A) A schematic representation of the relationship between the effective reproduction number (${R_e}$) and the time at which an individual acquire infection (${\tau _j}$). Nodes with higher centrality are expected to have a smaller area under the curve of ${\tau _j}$ and ${R_e}$, indicating their potential of acquiring infection earlier in an outbreak. (B) Informed by historical available observations of individuals (A, B, C, and D) over ($\eta \ $= 2) seasons, the average historical vulnerability of of individual *j*,$\ < {F^\eta }{ >_j}$, is estimated from the historical observations of infection time. Individuals, ranked by two factors (years of records and average infection time) from first to fourth, are selected sequentially from top to bottom and from left to right in our proposed surveillance strategy. The red and blue bars denote the observed and average infection timing of individuals across two historical seasons (e.g. 1 and 2), respectively.

Let $\eta $ be the number of influenza seasons with EHR data, and ${R_e}( i )$ the effective reproduction number of influenza infections in each season $i = 1,2,.\ .\ .,\ \eta $. Let ${\eta _j}$ be the number of influenza seasons in which individual *j* has EHR records of influenza infections. Let $\tau _j^i$ be the time at which individual *j* acquires infection in influenza season *i*, according to the EHR records. With these definitions, we assess the expected risk of having an EHR record of influenza infection in any influenza season for individual *j* as

$$\begin{eqnarray*}
{\langle {F^{{\eta _j}}}\rangle _j} &=& \frac{1}{{{\eta _j}}}\ \ \left( {{R_e}\left( 1 \right)\tau _j^1 + {R_e}\left( 2 \right)\tau _j^2 + \cdot \cdot \cdot + {R_e}\left( {{\eta _j}} \right)\tau _j^{{\eta _j}}} \right)\nonumber\\
&=& \frac{1}{{{\eta _j}}}\ \mathop \sum \limits_{i\ = {\rm{\ }}1}^{{\eta _j}} {R_e}\left( i \right)\tau _j^i,
\end{eqnarray*}
$$

which essentially estimates individual *j*’s expected infection time over all influenza seasons. The node with higher eigenvector centrality (a measure of the influence of a node operationalizing the recursive idea that central nodes are those who have many central neighbors) has a smaller area under the curve of *τ_j_* and *R_e_* (Fig. 1).

**Fig. 2.**
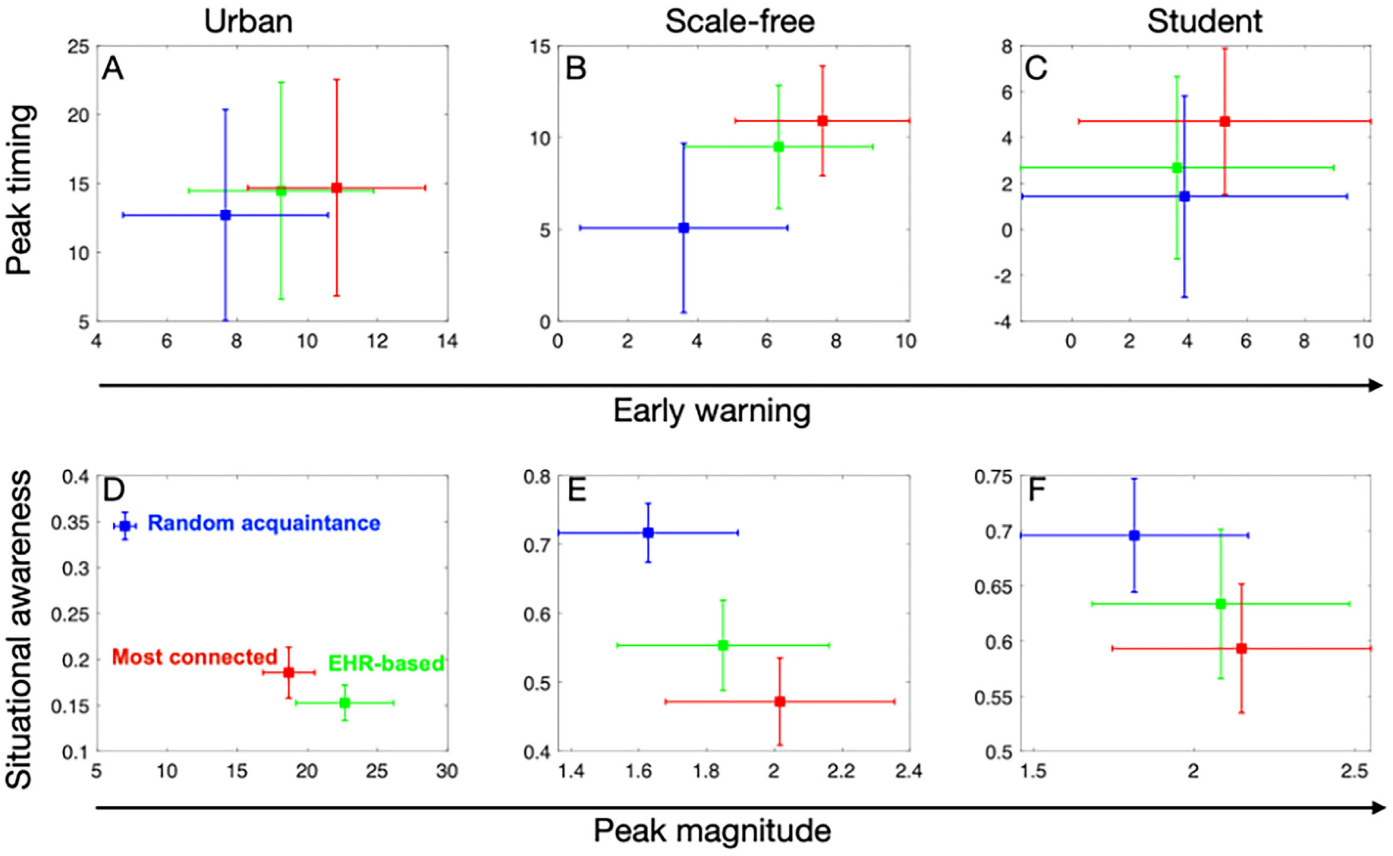
Performance of the most connected (red), random acquaintance (blue), and EHR-based (green) strategies. The EHR-based strategy here uses the EHR records obtained from five historical seasons as an example. In the upper panel, the horizontal and vertical axes present the early earning (days) and peak timing (days) measures for each strategy. In the bottom panel, the horizontal and vertical axes present the peak magnitude and situational awareness measures for each strategy. Panels from left to right correspond to the results using urban, scale-free, and student networks, respectively. In each panel, dots and error bars indicate the mean and standard deviation across 100 simulations of each strategy.

**Fig. 3.**
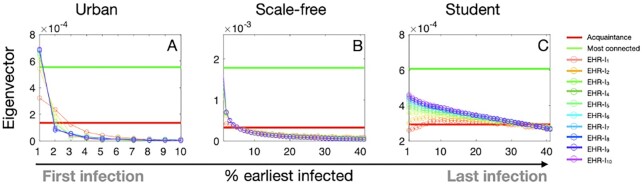
Average eigenvector centrality of surveillance nodes as a function of time (% earliest infected nodes). We compare the average eigenvector centrality of surveillance nodes identified from each strategy including random acquaintance strategy, most-connected strategy, and EHR-based strategies with increasing years of EHR influenza records. Panels from left to right correspond to the results using urban, scale-free, and student networks, respectively. The horizontal axis, the timeline, denotes % earliest infected nodes. Each strategy identifies a collection of surveillance nodes. For each $EHR$-${I_5}$ strategy, we run 100 simulations. For example, a simulation of $EHR$-${I_5}$, uses the first five sequential influenza-like simulations to select the surveillance nodes. We evaluate the average eigenvector centrality of 1% nodes over the horizontal axis with each step as 0.01. We observe that EHR-based performs better with increasing historical outbreaks involved. The average eigenvector centrality of surveillance subset EHR-based with shorter history, lays between that of the acquaintance and most connected strategies.

As in ref. (7), we consider the sentinels of surveillance nodes as the top 1% of individuals with highest expected risks of influenza infections over all seasons. Let EHR-I_η_ denote the EHR-based strategy using *η* influenza seasons of EHR records. We test the surveillance performance with *η* increasing from 1 to 10 seasons of EHR records. Our main analysis uses EHR-I_5_ with five seasons of EHR records (Fig. 2), because further increasing the number of EHR seasons *η* will give similar results (Fig. 3).

### Conventional network-based surveillance strategy

We compare our EHR-based strategy with two conventional network-based surveillance strategies, including (1) the *most-connected* strategy, which uses the top 1% of hub individuals with the highest numbers of network connections, and (2) the *random acquaintance* strategy, which first randomly selects 1% of individuals in the network and then uses one random acquaintance of each randomly selected individual as the surveillance node. As in ref.^7^, we use 1% of individuals in the network as the surveillance nodes.

### Simulation settings

We simulate the spread of SARS-CoV-2 and seasonal influenza in contact networks, in which nodes denote individuals and edges denote physical contacts. We use three different networks, including the *urban network* of public wireless network usage (19), *scale-free network* built with Barabási–Albert (BA) algorithm (20), and *students network* of class attendance (7). The degree distribution has a power–law pattern in the scale-free and urban networks and has a Poisson-like pattern in the students network. Details of these networks are specific in Methods. We use a susceptible-exposed-infectious-recovered (SEIR) epidemic model to describe the historical spread of seasonal influenza, and use an susceptible-exposed-asymptomatic-symptomatic-recovered (SEAYR) model to describe the contemporary spread of SARS-CoV-2 (Methods). We use the stochastic chain-binomial approach to simulate the spread of these epidemics in contact networks.

### Four criteria to evaluate the performance of surveillance strategies

We consider the disease prevalence of SARS-CoV-2 as the proportion of infected, individuals including exposed, asymptomatic, and symptomatic individuals at a given time. Based on refs. (7, 21, 22), we use the following four criteria to evaluate the performance of each surveillance strategy in monitoring the SARS-CoV-2 epidemic:

1. *Early warning*. Let $t_\mu ^{EP}$ be the time at which the disease prevalence in the entire population (EP) reaches a predefined threshold *μ*, and $t_\mu ^{SG}$ the time at which the disease prevalence in the SG reaches the same threshold *μ*. We consider *μ* = 1%. The early warning criterion measures the time lag: $t_\mu ^{EP} - t_\mu ^{SG}$.
2. *Peak timing*. Let $t_{{\rm{peak}}}^{EP}$ be the time at which the disease prevalence in the EP reaches the peak, and $t_{{\rm{peak}}}^{SG}$ the time at which the disease prevalence in the SG reaches the peak. The peak timing criterion measures the time lag: $t_{{\rm{peak}}}^{EP} - t_{{\rm{peak}}}^{SG}$.
3. *Peak magnitude*. Let $r_{{\rm{peak}}}^{EP}$ be the peak value of the disease prevalence in the EP, and $r_{{\rm{peak}}}^{SG}$ the peak value of the disease prevalence in the SG. The peak magnitude criterion measures the ratio: $r_{{\rm{peak}}}^{EP}/r_{{\rm{peak}}}^{SG}$.
4. *Situational awareness*. The complement of the normalized mean absolute error (MAE) of the time series of distance prevalence between the SG and EP:
  $$\begin{equation*}
1 - \mathop {\min }\limits_\lambda \frac{{\sum\nolimits_t {|{x_t} - {y_{t + \lambda }}|} }}{{\sum\nolimits_t {|{x_t} - {y_{t + \lambda }}|} }},
\end{equation*}
$$

which is minimized over possible time lags λ (7). Here, *x_t_* and *y_t_* denote the disease prevalence of the simulated SARS-CoV-2 epidemics in the EP and SG at time *t*, respectively.

## Main findings

The EHR-based strategy outstrips the random acquaintance strategy in almost all four evaluation criteria. The performance of our EHR-based strategy is comparable to that of the most connected strategy (Fig. 2). In heterogeneous networks including the urban and scale-free networks, the EHR-based strategy and the most-connected strategy both provide good performance in the surveillance tasks of early warning and peak timing. In all tested networks, the peak magnitude predicted by our EHR-based strategy is much closer to that predicted by the most-connected strategy as compared to the random acquaintance strategy.

Specifically, in the urban network (Fig. 2A and D), on average, the EHR-based strategy can trigger an early warning 9.8 days before the whole population reaches a predefined threshold, 24% faster than the random acquaintance strategy (7.9 days on average). On average, it has a peak timing, peak magnitude, and situational awareness of 13.5 days, 22.38 days, and 0.15, respectively. When compared with the random acquaintance, the EHR-based strategy shows a peak timing 14% higher, an overestimation of the peak magnitude of 3.2 times and 44% decrease in situational awareness. In the scale-free network (Fig. 2B and E), the EHR-based strategy has an early warning, peak timing, peak magnitude, and situational awareness of 6.3 days, 8.9 days, 1.82, and 0.54, respectively. On average, the performance of our strategy shows a 75% improvement in early warning, 109% overestimation in peak timing, 1.1 times in peak magnitude, and 75% decrease in situational awareness compared with random acquaintance. In the student network (Fig. 2C and F), the EHR-based strategy has early warning, peak timing, peak magnitude, and situational awareness of 3.61 days, 2.68 days, 2.08, and 0.63, respectively. When compared with the random acquaintance, it has 7% less in early warning, 87% overestimation in peak timing, 1.15 times in peak magnitude, and 9% decrease in situational awareness.

To explain the performance of the surveillance strategies, we explore the subsets of individuals selected by these surveillance strategies in terms of their eigenvector centrality. Figure 3 suggests that the EHR-based strategy appears to select nodes with higher eigenvector centralities. Increasing the number of influenza seasons used in the EHR-based strategy facilitates the identification of central nodes in the network. Lastly, EHR-based selection of nodes, is more similar to the selection made by the most-connected strategy, as we increase the number of historical outbreaks (Figure S4, Supplementary Material).

## Discussion

From contact network epidemiology, we know that central nodes are at higher risk of being infected early in an epidemic and can, thus be identified as being disproportionately represented among the previously infected in seasonal diseases (for instance, influenza, Chlamydia, and Lyme disease) (15). Building on the availability of EHR systems, we propose a novel surveillance strategy; selection based on historical records of infection, which can be implemented in the context of sentinel placement for COVID-19 surveillance, denoted as the EHR-based strategy. The advantage of this approach is that if the contact structure (or risk behavior based on the connectivity of individuals) is persistent enough, then it will, on average, be reflected by the history of infection of each individual. Thus, this method exploits the effect of contact structure without the knowledge of the network itself—which is both difficult and relies on the assumption that the structures are persistent.

Through epidemic simulations in static contact networks, we found that this novel strategy can accelerate the epidemic outbreak detection process, competing with the other static-network strategies in terms of practicality and early warning. To assess the centrality dynamics of nodes selected by the EHR-based strategy, we calculate and compare (computationally and theoretically) the eigenvector centrality of the nodes selected by each strategy on both empirical and synthetic networks. We find that our proposed surveillance strategy is competitive when compared with other strategies and depends on the number of historical outbreaks and the public health objective.

We studied the relationship between the selection of nodes using the EHR-based strategy and the optimal theoretical surveillance subsets (see Method). Following percolation theory on networks (7, 23), where an SEIR infectious disease is spreading, we calculate analytically the optimal surveillance subset. We show that the selection of nodes in the surveillance subset when applying the EHR-based strategy, and the optimal theoretical selection of nodes (those with the highest eigenvector centrality) tend to be similar, as the number of historic records increases.

In the context of an actual new emerging or reemerging infectious disease (e.g. COVID-19), the EHR-based strategy can be applied using historical records of a different (related) disease. The ranking of individuals can be learned from the knowledge of other infectious diseases belonging to the same spatial scenario, concurrently or sequentially. For example, the transmission dynamics learned from the surveillance of seasonal influenza can be used to estimate the outbreak risk of varicella in Taiwan (24).

Although we believe our qualitative results are robust and implementable, we need to address a few simplifying assumptions. First, our model does not account for the reinfection of influenza within a single flu season. The temporal cross-strain immunity is estimated with a short duration according to the real-world data (e.g. 42 days in the US (25)). However, if the circulating strain remains the same during two consecutive influenza seasons, the prior immunity gained in the past season may protect the previously infected individuals from the reinfection of the same strain. To reduce this potential bias, we suggest excluding these years. Second, our proposed strategy identifies a small proportion of the population as surveillance nodes for early detection of new outbreaks. Our identified surveillance individuals may not be representative of the EP, and hence may not be suitable for other surveillance purposes such as estimating the final attack rate or population prevalence. Third, the accessibility of EHR data could be limited by privacy-related restrictions, which could narrow the applications of the method. Fourth, the basic reproduction number may not be estimated directly in an influenza season. However, it could be approximated using the effective reproduction number, vaccine coverage, and vaccine efficacy. Fifth, it is possible that not all infected individuals will have their influenza records registered. We perform a sensitivity analysis by reducing the probability of seeking treatment and having an influenza record in the EHR for each infected individual *P* (health-seeking) from 75% to 25% (Figures S1 to S3, Supplementary Material). We find that the EHR-based strategy does not work well when *P* (health-seeking) reduces to 25%.

We conclude that the proposed EHR-based strategy for sentinel surveillance selection is competitive with other existing surveillance strategies in networks. This strategy, in general, could prove useful to public health policy makers, by offering a practical and robust alternative without the knowledge of individual contact behaviors, especially when a long enough history of EHR in public health systems is available. In this study, we provide a new method for surveillance of populations, which can also be used synergistically with network-based strategies. Additionally, our EHR-based strategy could be extended to consider the case of targeted testing and targeted vaccination.

## Materials and Methods

### Modeling the historical spread of seasonal influenza in contact networks

We simulate epidemic outbreaks using a stochastic chain-binomial model in contact networks with nodes as individuals and edges as interpersonal physical contacts. The degree of a node is the number of other nodes connected to it via its edges.

For seasonal influenza, each individual has four states: susceptible (S), exposed (E), infectious (I), or recovered (R). The transmission rate of the disease is *β*. Node *i* will remain exposed for 1/*σ* days and infectious for 1/*γ* days, after which it will recover. The basic reproduction number of a disease, denoted *R*_0_, demonstrates the expected number of secondary infections caused by a single infection in an entirely susceptible population, commonly used to indicate the epidemic growth rate, which is approximately equal to the effective reproduction number (*R_e_*, the average number of secondary cases per infectious case in a population made up of both susceptible and nonsusceptible hosts) given most individuals are susceptible in our simulations. We fix *σ* and *γ* for every simulation to 4 and 7 days, respectively, within the range of estimates for common respiratory diseases, including influenza (26). The disease prevalence is counted as the number of people in E over time. Let *R*_0_ follow the distribution of Triangular(1.12, 1.25, and 1.33) according to the seasonal influenza epidemics over countries from 2000 to 2011 (27).

### Modeling the contemporary spread of SARS-CoV-2 in contact networks

To test the performance of the proposed strategy on COVID-19 scenarios, each individual has five states: susceptible (S), exposed (E), asymptomatic (A), symptomatic (Y), or recovered (R). Node *i* will remain exposed for 1/*σ_C_* days, after which it will become infectious for 1/*γ_C_* days as asymptomatic and symptomatic with probabilities of 1–*p_sym_* and *p_sym_*, respectively, after which it will recover. The infectiousness of asymptomatic individuals is likely to be different from those with symptoms, perhaps by shedding lower quantities of the infectious agent and having more potential contacts with others (28). And asymptomatic individuals have been considered with obvious differences of infectiousness, in contrast with others with symptoms (29). The relative infectiousness of an asymptomatic individual (A) is *ω*. The transmission rate of the disease is *ωβ_C_* and *β_C_* for asymptomatic and symptomatic states, respectively. We fix *R*_0_, *σ_C_, γ_C_, ω*, and *p_sym_* for every simulation to 2.5 (1, 30), 1/5 days (31), 1/2.5 days (32), 0.5 (32), and 0.75 (33), respectively. In contact networks (with arbitrary degree distributions but random in any other aspect), *R*_0_ correlates with the transmission rate, as (34)

(1) $$\begin{equation*}
{R_0} = \beta \left( {\frac{{\langle {k^2}\rangle - \langle k\rangle }}{{\langle k\rangle }}} \right),
\end{equation*}
$$

where $\langle k\rangle $ and $\langle {k^2}\rangle $ denote the mean and mean square of degree. Following the static-network strategies' evaluation (7), given a specific *R*_0_, we use Eq. 1 to solve the corresponding transmission rate *β* for influenza. For COVID-19 scenarios, we estimate *β_C_* by $\frac{\beta }{{{p_{sym}} + (1 - \omega {p_{sym}})}}$. We start simulations in scale-free and student networks with one randomly sampled seed to be exposed, while urban networks have 100 seeds. We investigate various epidemic outbreaks in networks to reflect the transmission variation of infectious disease (7).

### Contact network datasets

In this study, we consider the spread of epidemics in a networked population, in which individuals in the population are connected through contact networks (7, 35–37). Following ref.^7^, we use the following three networks in which the interpersonal contacts are described by unweighted connections. We consider these three networks to explore the influence of their distinct topological properties.

1. *Urban network*. This colocation network consists of 103,425 users (i.e. nodes) of the Île Sans Fil free public wireless network in Montreal, Canada. In this network, the connections represent the concurrent hotspot usage (19).
2. *Scale-free network*. This topologically heterogeneous network is generated using the seminal BA algorithm (20).
3. *Student network*. This network consists of 4,634 students (i.e. nodes) of the Engineering Department from the Universidad de Los Andes in Mérida-Venezuela. In this network, the connections indicate that a group of students shared at least one class during the fall 2008 semester (7).

The degree distribution shows a power–law pattern in the scale-free and urban networks (20, 19), and shows a Poisson-like pattern in the students network. Furthermore, the urban network has a strong community structure (19).

### Centrality dynamics of our strategy

We use the analytical method developed in ref.^7^ to study the relationship between the surveillance nodes selected in our EHR-based strategy and the analytically derived optimal set of surveillance nodes. We use an SEIR-like epidemic model to describe the spread of seasonal influenza in a network of size *N*, in which $\beta $ denotes the transmission rate per infectious contact and $\gamma $ denotes the recovery rate. According to the percolation theory developed in refs. (7, 23), during the initial outbreak, the probability that each node acquires infection at time *t* is approximated as

(2) $$\begin{equation*}
x(t) = {e^{(\beta \kappa - \gamma ){t_\upsilon }}},
\end{equation*}
$$

where *κ* is the leading eigenvalue of the adjacency matrix of the network, and *v* the corresponding eigenvector. This formula suggests that nodes with larger eigenvectors are more likely to have an earlier infection. Therefore, ref. 7 suggests that the optimal set of surveillance nodes need to include those nodes with highest eigenvector centralities.

Let *M*_EHR-I_ be the set of surveillance nodes used in our EHR-based strategy, which are determined by historical EHR influenza infection records. During the initial outbreak, the eigenvector centralities for the surveillance nodes in our EHR-based strategy is given by

$$\begin{equation*}
{\rm{\ }}{c_{EHR - I}} = \frac{{v \cdot {1_{EHR - I}}}}{{{M_{EHR - I}}}}{\rm{\ }} \approx {\rm{\ }}\frac{{v \cdot x\left( t \right)}}{{{M_{EHR - I}}}} = \frac{{{e^{\left( {\beta \kappa - \gamma } \right)t}}{\upsilon ^2}}}{{{M_{EHR - I}}}}\
,
\end{equation*}
$$

where ${1_{EHR - I}}$ is an indicator vector with elements being 1 if the corresponding nodes are chosen as surveillance nodes in the EHR-based strategy and vice versa. Our EHR-based surveillance strategy can identify high-risk nodes with largest eigenvector centralities, as indicated by the large average eigenvector centralities ${c_{EHR - I}}$ for nodes acquiring earliest infections (Fig. 3).

Nodes are infected and selected as time advances. During the initial outbreak, ${c_{EHR - I}}$ is increasing with time via selecting nodes with high eigenvector centralities. After that, low eigenvector centrality nodes will be infected and selected (7), and thus, the EHR-based SG tends to be the optimal by selecting those nodes infected earlier than other nodes, which tends to have higher eigenvector centrality.

Following ref.^7^, let ${\tau _{EHR - I}}$ and $\tau $ be the times at which the EHR-based SG and the other SG with size *M* reach the same prevalence threshold *p*. Let $1$ be the indicator vector of dimension *N*, denoting the nodes selected by the EHR-based strategy. As thus,

$$\begin{equation*}
p\ = \frac{{{e^{\left( {\beta \kappa - \gamma } \right){\tau _{EHR - I}}}}v \cdot {1_{EHR - I}}}}{{{M_{EHR - I}}}}{\rm{\ }} = \frac{{{e^{\left( {\beta \kappa - \gamma } \right)\tau }}v \cdot 1}}{{\rm{M}}}.
\end{equation*}
$$

The timing of early warning achieved between the two SGs of the EHR-based and the other SGs, denoted $\Delta \ {t_{EHR - I}} = \ \tau - {\tau _{EHR - I}}$, implies

$$\begin{equation*}
\Delta \ {t_{EHR - I}} = \frac{1}{{\beta \kappa - {\gamma _{}}}}{\rm{\ ln}}\left( {\frac{{{c_{EHR - I}}}}{c}} \right),
\end{equation*}
$$

where ${c_{EHR - I}} = \ v \cdot {1_{EHR - I}}/{M_{EHR - I}}$ and $c\ = \ v \cdot {1_{EHR - I}}/M$ are the average eigenvector centralities in the two surveillance subsets, respectively. The early warning timing between the other SG and the EHR-based surveillance subset is determined by the ratio of their average eigenvector centralities. Therefore, during the initial outbreak, high eigenvector centrality nodes become infected with higher probability. After this initial regime, where most nodes with the highest eigenvector centralities have been infected, the infection spreads to nodes in the periphery of the network, i.e. nodes with low rankings of eigenvector centrality. Therefore, the average eigenvector centrality of all infected nodes decreases smoothly as time increases.

Considering an individual *j* in season ${\eta _j}$, the probability, $x({t_j},\ {\eta _j})$, of being infected at time ${t_j}$ is proportional to

$$\begin{equation*}
\theta ({t_j},{\eta _j}){\rm{\ }} = {\rm{\ log\ }}\left( {\frac{{x({t_j},{\eta _j})}}{{\gamma v}}} \right) = {R_0}{\rm{\ }}\left( {{\eta _j}} \right)\kappa {t_j} - {t_j}.
\end{equation*}
$$

The ratio of $\theta ({t_j},\ {\eta _j})$ in two seasons (*i* and *j*) implies

$$\begin{equation*}
{\rm{\ }}\frac{{\theta ({t_i},i)}}{{\theta ({t_j},j)}} = \frac{{{R_0}\left( i \right)\kappa {t_i} - {t_i}}}{{{R_0}\left( j \right)\kappa {t_j} - {t_j}}}{\rm{\ }} \sim \frac{{{R_0}\left( i \right){t_i}}}{{{R_0}\left( j \right){t_j}}} \sim \frac{{{R_e}\left( i \right){t_i}}}{{{R_e}\left( j \right){t_j}}}.
\end{equation*}
$$

Hence, in our proposed strategy, the historical vulnerability of an individual is a combination of $\tau _j^i$ and ${R_0}( i )$ (or ${R_e}( i )$) the time of individual *j* in season *i*.

## Code Availability

All codes used in this study can be accessed upon publication of this article at https://github.com/ZhanweiDU/earlydetection.

## Classification

Major category: biological sciences; minor category: medical sciences.

## Funding

Financial support was provided by the AIR@InnoHK administered by the Innovation and Technology Commission, the Collaborative Research Fund (project no. C7123-20G) of the Research Grants Council of the Hong Kong SAR Government, the National Natural Science Foundation of China (grant nos. 72104208, 41930104, and 41971341), the Research Program of Shenzhen S&T Innovation Committee Project (grant no. JCYJ20210324093600002), the US National Institutes of Health (grant no. R01 AI151176) and the CDC COVID Supplement (grant no. U01IP001136). P.H. was supported by JSPS KAKENHI grant no. JP21H04595.

## Supplementary Material

pgac038_Supplemental_File

## Data Availability

The data that support the findings of this study are available from Crawdad (38) and ref. 7.

## References

[1] Li Q , et al. 2020. Early transmission dynamics in Wuhan, China, of novel coronavirus–infected pneumonia. N Engl J Med. 382:1199–1207. 31995857 10.1056/NEJMoa2001316 PMC7121484

[2] WHO . 2020. WHO declares a pandemic of coronavirus disease covid-19. The Washington Post. March 25, 2020.

[3] Center for Systems Science and Engineering (CSSE) . COVID-19 dashboard by the Center for Systems Science and Engineering (CSSE). Baltimore (MD): Johns Hopkins University (JHU). September 28, 2020.

[4] Desjardins MR , Hohl A, Delmelle EM. 2020. Rapid surveillance of COVID-19 in the United States using a prospective space-time scan statistic: detecting and evaluating emerging clusters. Appl Geogr. 118:102202. 32287518 10.1016/j.apgeog.2020.102202 PMC7139246

[5] Desjardins MR . 2020. Syndromic surveillance of COVID-19 using crowdsourced data. Lancet Region Health West Pac. 4:100024. 10.1016/j.lanwpc.2020.100024 PMC7563097 34013214

[6] Yoneoka D , et al. 2020. Large-scale epidemiological monitoring of the COVID-19 epidemic in Tokyo. Lancet Region Health West Pac. 3:100016. 10.1016/j.lanwpc.2020.100016 PMC7546969 34173599

[7] Herrera JL , Srinivasan R, Brownstein JS, Galvani AP, Meyers LA. 2016. Disease surveillance on complex social networks. PLoS Comput Biol. 12:e1004928. 27415615 10.1371/journal.pcbi.1004928 PMC4944951

[8] Volz E . 2008. SIR dynamics in random networks with heterogeneous connectivity. J Math Biol. 56:293–310. 17668212 10.1007/s00285-007-0116-4 PMC7080148

[9] Meyers LA , Newman MEJ, Pourbohloul B. 2006. Predicting epidemics on directed contact networks. J Theor Biol. 240:400–418. 16300796 10.1016/j.jtbi.2005.10.004

[10] Meyers LAl , Newman MEJ, Martin M, Schrag S. 2003. Applying network theory to epidemics: control measures for *Mycoplasma pneumoniae* outbreaks. Emerg Infect Dis. 9:204–210. 12603991 10.3201/eid0902.020188 PMC3369603

[11] Meyers LA , Pourbohloul B, Newman MEJ, Skowronski DM, Brunham RC. 2005. Network theory and SARS: predicting outbreak diversity. J Theor Biol. 232:71–81. 15498594 10.1016/j.jtbi.2004.07.026 PMC7094100

[12] Newman MEJ . 2002. Spread of epidemic disease on networks. Phys Rev E Stat Nonlin Soft Matter Phys. 66:016128. 12241447 10.1103/PhysRevE.66.016128

[13] Bai Y , et al. 2017. Optimizing sentinel surveillance in temporal network epidemiology. Sci Rep. 7:4804. 28684777 10.1038/s41598-017-03868-6 PMC5500503

[14] Christakis NA , Fowler JH. 2010. Social network sensors for early detection of contagious outbreaks. PLoS ONE. 5:e12948. 20856792 10.1371/journal.pone.0012948 PMC2939797

[15] Yamin D , et al. 2014. An innovative influenza vaccination policy: targeting last season's patients. PLoS Comput Biol. 10:e1003643. 24851863 10.1371/journal.pcbi.1003643 PMC4031061

[16] Park YJ , et al. 2020. Contact tracing during coronavirus disease outbreak, South Korea, 2020. Emerg Infect Dis. 26:2465–2468. 32673193 10.3201/eid2610.201315 PMC7510731

[17] Han E , et al. 2020. Lessons learnt from easing COVID-19 restrictions: an analysis of countries and regions in Asia Pacific and Europe. Lancet. 396:1525–1534. DOI: 10.1016/S0140-6736(20)32007-9. 32979936 PMC7515628

[18] Holme P . 2017. Three faces of node importance in network epidemiology: exact results for small graphs. Phys Rev E. 96:062305. 29347435 10.1103/PhysRevE.96.062305 PMC7217518

[19] Hoen AG , et al. 2015. Epidemic wave dynamics attributable to urban community structure: a theoretical characterization of disease transmission in a large network. J Med Internet Res. 17:e169. 26156032 10.2196/jmir.3720 PMC4526984

[20] Barabasi AL , Albert R. 1999. Emergence of scaling in random networks. Science. 286:509–512. 10521342 10.1126/science.286.5439.509

[21] Thompson WW , Comanor L, Shay DK. 2006. Epidemiology of seasonal influenza: use of surveillance data and statistical models to estimate the burden of disease. J Infect Dis. 194 Suppl 2:S82–S91. 17163394 10.1086/507558

[22] Rath TM , Carreras M, Sebastiani P. 2003. Automated detection of influenza epidemics with Hidden Markov Models. In: Advances in intelligent data analysis V. Berlin, Heidelberg: Springer. p. 521–532.

[23] Newman M . 2010. Networks: an introduction. Oxford: OUP.

[24] Yang B , Pei H, Chen H, Liu J, Xia S. 2017. Characterizing and discovering spatiotemporal social contact patterns for healthcare. IEEE Trans Pattern Anal Mach Intell. 39:1532–1546. 27608452 10.1109/TPAMI.2016.2605095

[25] Fox SJ , Miller JC, Meyers LA. 2017. Seasonality in risk of pandemic influenza emergence. PLoS Comput Biol. 13:e1005749. 29049288 10.1371/journal.pcbi.1005749 PMC5654262

[26] De Serres G , et al. 2010. Contagious period for pandemic (H1N1) 2009. Emerg Infect Dis. 16:783–788. 20409367 10.3201/eid1605.091894 PMC2954014

[27] Biggerstaff M , Cauchemez S, Reed C, Gambhir M, Finelli L. 2014. Estimates of the reproduction number for seasonal, pandemic, and zoonotic influenza: a systematic review of the literature. BMC Infect Dis. 14:480. 25186370 10.1186/1471-2334-14-480 PMC4169819

[28] McEvoy D , et al. 2021. Relative infectiousness of asymptomatic SARS-CoV-2 infected persons compared with symptomatic individuals: a rapid scoping review. BMJ Open. 11:e042354. 10.1136/bmjopen-2020-042354 PMC8098293 33947725

[29] Johansson MA , et al. 2021. SARS-CoV-2 transmission from people without COVID-19 symptoms. JAMA Netw Open. 4:e2035057. 33410879 10.1001/jamanetworkopen.2020.35057 PMC7791354

[30] Sugishita Y , Kurita J, Sugawara T, Ohkusa Y. 2020. Preliminary evaluation of voluntary event cancellation as a countermeasure against the COVID-19 outbreak in Japan as of 11 March, 2020. medRxiv. 10.1101/2020.03.12.20035220. PMC7751859 33347444

[31] Backer JA , Klinkenberg D, Wallinga J. 2020. Incubation period of 2019 novel coronavirus (2019-nCoV) infections among travellers from Wuhan, China, 20-28 January 2020. Euro Surveill. 25:2000062. 10.2807/1560-7917.ES.2020.25.5.2000062 PMC7014672 32046819

[32] Aleta A , et al. 2020. Modelling the impact of testing, contact tracing and household quarantine on second waves of COVID-19. Nat Hum Behav. 4:964–971. 32759985 10.1038/s41562-020-0931-9 PMC7641501

[33] Nishiura H , et al. 2020. Estimation of the asymptomatic ratio of novel coronavirus infections (COVID-19). Int J Infect Dis. 94:154–155. 32179137 10.1016/j.ijid.2020.03.020 PMC7270890

[34] Meyers L . 2007. Contact network epidemiology: bond percolation applied to infectious disease prediction and control. Bull Am Math Soc. 44:63–86.

[35] Masuda N , Holme P. 2017. Temporal network epidemiology. Singapore: Springer.

[36] Pastor-Satorras R , Castellano C, Van Mieghem P, Vespignani A. 2015. Epidemic processes in complex networks. Rev Mod Phys. 87:925–979.

[37] Wang L , Li X. 2014. Spatial epidemiology of networked metapopulation: an overview. Chin Sci Bull. 59:3511–3522. 32214746 10.1007/s11434-014-0499-8 PMC7088704

[38] Lenczner M , Hoen AG. 2015. CRAWDAD dataset ilesansfil/wifidog (v. 2015-11-06). 10.15783/C7H883, last accessed date Apr. 29, 2022.

