# Supplementary material for: Optimizing COVID-19 surveillance using historical electronic health records of influenza infections
Source: PNAS Nexus. 2022 Apr 14;1(2):pgac038. doi: 10.1093/pnasnexus/pgac038 (PMC9170911; doi:10.1093/pnasnexus/pgac038)
Supplement: pgac038_Supplemental_File [file pgac038_supplemental_file.pdf]

Supplementary Material

Optimizing COVID-19 surveillance using historical electronic health records of influenza infections

Zhanwei Du[1,2,3,4†], Yuan Bai[2,3†], Lin Wang[5†], Jose L. Herrera-Diestra[4,6†], Zhilu Yuan[7†], Renzhong Guo[7], Benjamin J Cowling[2,3], Lauren Ancel Meyers[4*], Petter Holme[8,9*]

[1] The University of Hong Kong Shenzhen Institute of Research and Innovation, Shenzhen, China

[2] World Health Organization Collaborating Centre for Infectious Disease Epidemiology and Control, School of Public Health, University of Hong Kong, Hong Kong SAR, China.

[3] Laboratory of Data Discovery for Health, Hong Kong Science and Technology Park, Hong Kong Special Administrative Region, Hong Kong SAR, China

[4] The University of Texas at Austin, Austin, Texas 78712, The United States of America

[5] University of Cambridge, Cambridge CB2 3EH, UK

[6] Department of Biology, The Pennsylvania State University, University Park, PA, USA

[7] Research Institute for Smart Cities, School of Architecture and Urban Planning, Shenzhen University, Shenzhen, China

[8] Department of Computer Science, Aalto University, Espoo, Finland

[9] Center for Computational Social Science, Kobe University, Kobe, Japan

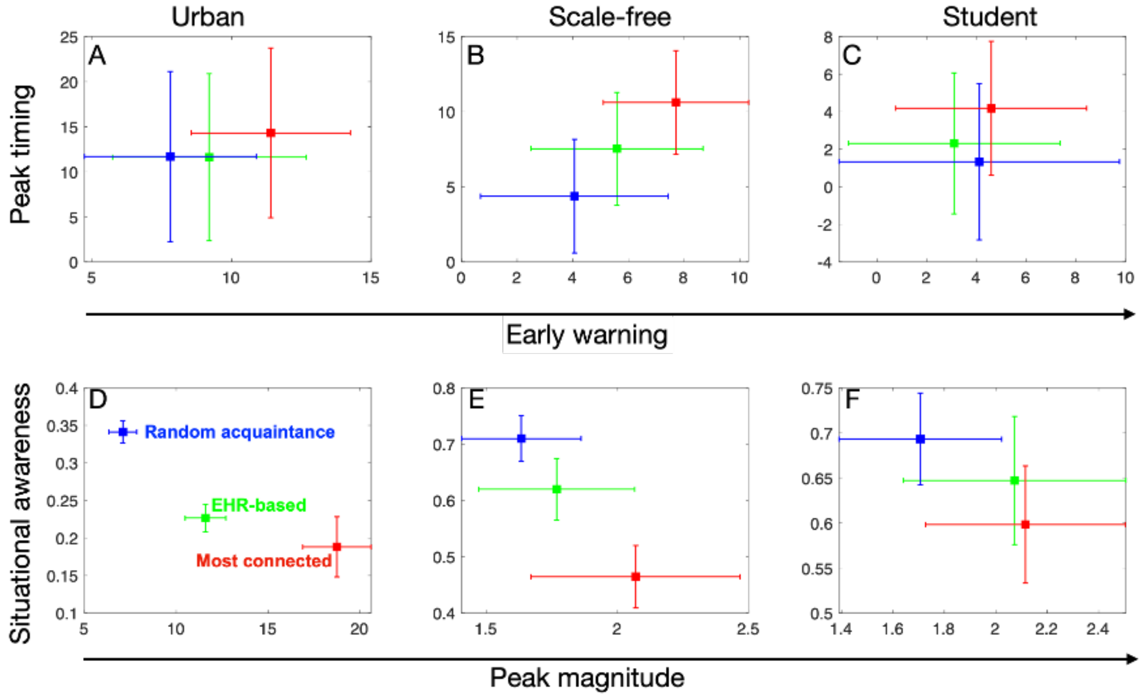

Figure S1. Performance of most connected (red), random acquaintance (blue), and EHR-based with 5 historical outbreaks (green) strategies with respect to four objectives: the early warning (days), the peak timing (days), the peak magnitude and the situational awareness. We assume an infected individual has a 75% probability of seeking treatment and having the electronic health record. Points and error bars indicate mean and standard deviation in performance over 100 simulations, respectively. Specifically, we run 100 simulations in urban, scale-free and student networks.

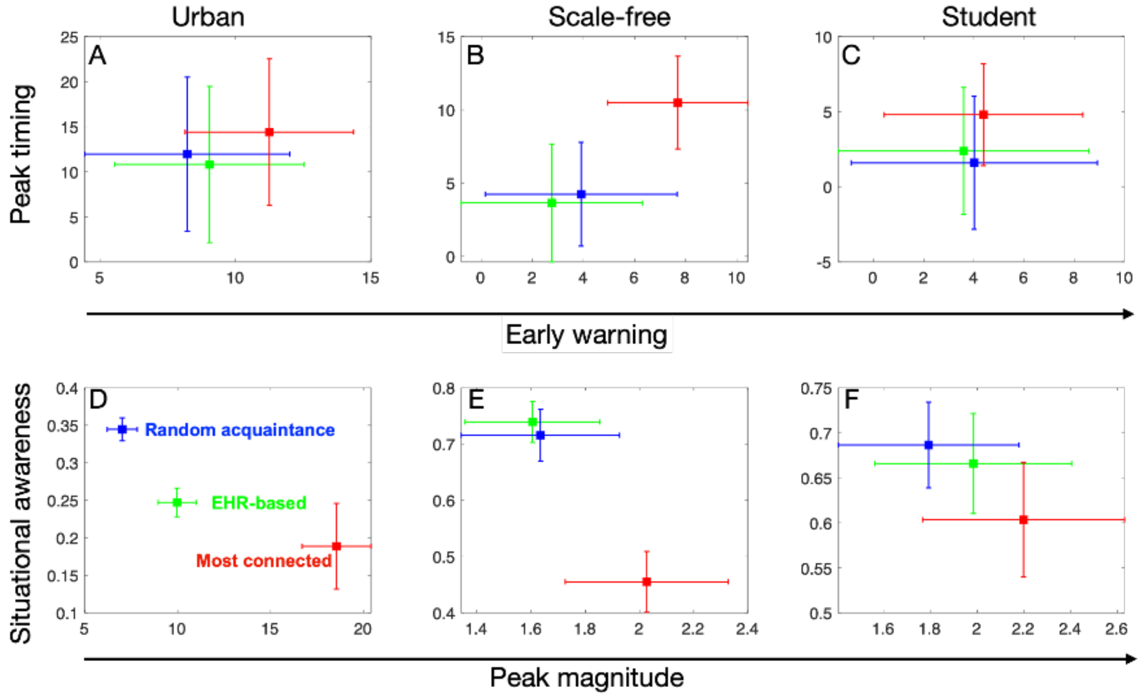

Figure S2. Performance of most connected (red), random acquaintance (blue), and EHR-based with 5 historical outbreaks (green) strategies with respect to four objectives: the early warning (days), the peak timing (days), the peak magnitude and the situational awareness. We assume an infected individual has a 50% probability of seeking treatment and having the electronic health record. Points and error bars indicate mean and standard deviation in performance over 100 simulations, respectively. Specifically, we run 100 simulations in urban, scale-free and student networks.

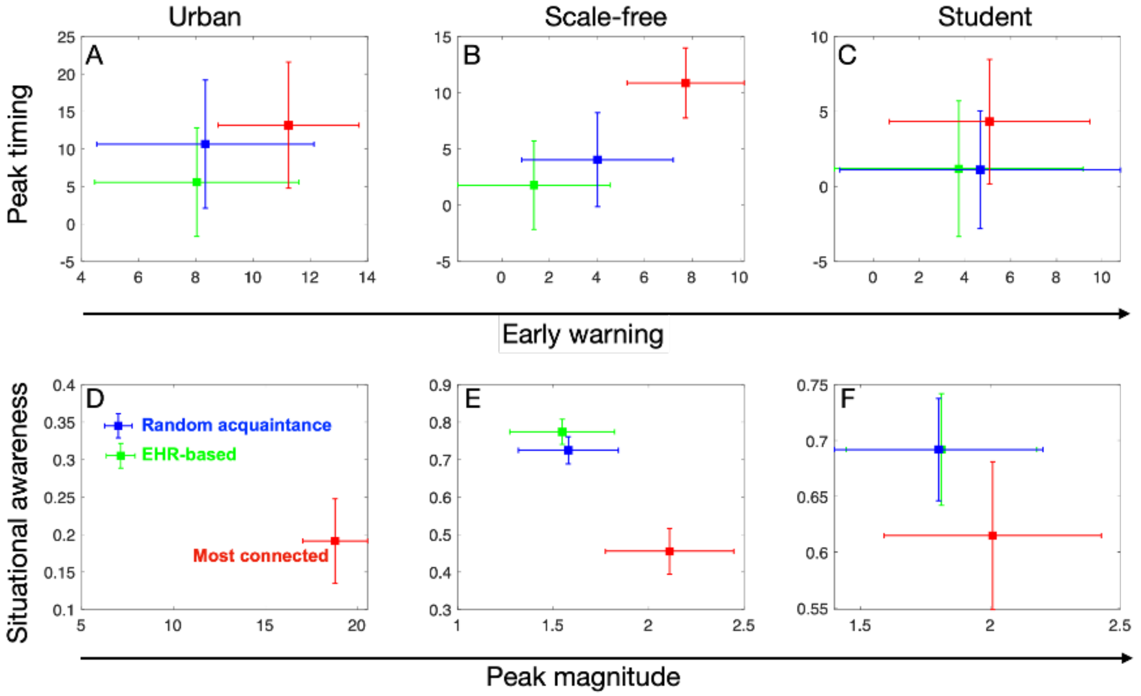

Figure S3. Performance of most connected (red), random acquaintance (blue), and EHR-based with 5 historical outbreaks (green) strategies with respect to four objectives: the early warning (days), the peak timing (days), the peak magnitude and the situational awareness. We assume an infected individual has a 25% probability of seeking treatment and having the electronic health record. Points and error bars indicate mean and standard deviation in performance over 100 simulations, respectively. Specifically, we run 100 simulations in urban, scale-free and student networks.

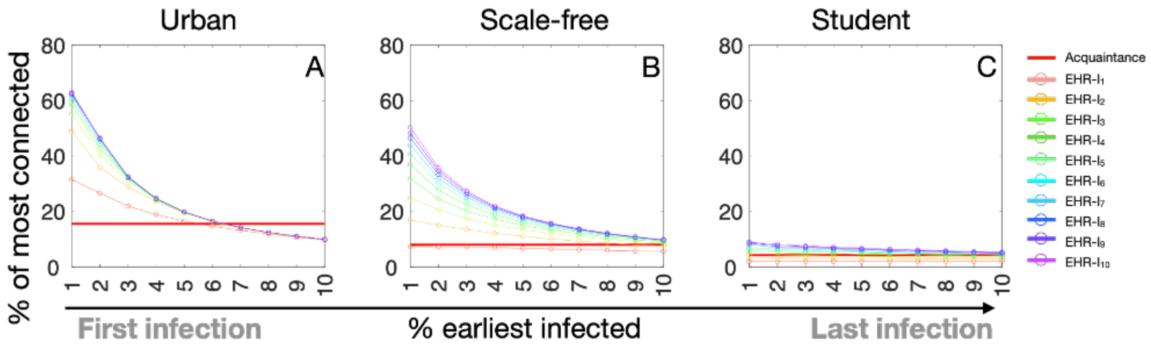

Figure S4. Overlapped surveillance nodes between the EHR-based (or random acquaintance) strategy and the most connected strategy as the COVID-19 epidemic unfolds. The EHR-based strategy ($\mathrm{EHR\text{-}I}_n$) considers the length of health records (*n*) from 1 year to 10 years. For example, the $\mathrm{EHR\text{-}I}_5$ strategy uses the first 5 sequential influenza seasons to learn the mean infection time of each node. Each panel from left to right provides the comparative results with the use of urban, scale-free or student networks, respectively. In each panel, each colored curve demonstrates the change in the percentage of the overlapped surveillance nodes between each given strategy and the most connected strategy as the increase in the infected nodes. In each panel, the horizontal axis describes the increase in the infected nodes as the increasing percentage of the earliest infected nodes. For each $\mathrm{EHR\text{-}I}_n$ strategy, we run 100 simulations. We estimate the percentage of nodes surveilled on average, which are also in the surveillance by the strategy of most connected, which are still in the earliest % nodes over the horizontal axis with each step as 1%. The overlapped surveillance nodes between the EHR-based strategy and the most connected strategy have a substantial increase as the considered length of health (*n*) records increases.
